# Establishment of *Agrobacterium tumefaciens*-Mediated Transformation of *Cladonia macilenta*, a Model Lichen-Forming Fungus

**DOI:** 10.3390/jof7040252

**Published:** 2021-03-26

**Authors:** Rundong Liu, Wonyong Kim, Jaycee Augusto Paguirigan, Min-Hye Jeong, Jae-Seoun Hur

**Affiliations:** 1 Korean Lichen Research Institute, Sunchon National University, Suncheon 57922, Korea; lrdlichen@gmail.com (R.L.); jagpaguirigan@gmail.com (J.A.P.); minhye1962@gmail.com (M.-H.J.); 2 Department of Biological Sciences, College of Science, University of Santo Tomas, España Boulevard, Manila 1008, Philippines

**Keywords:** *Cladonia*, lichen-forming fungi, *Agrobacterium*, ATMT, genetic transformation

## Abstract

Despite the fascinating biology of lichens, such as the symbiotic association of lichen-forming fungi (mycobiont) with their photosynthetic partners and their ability to grow in harsh habitats, lack of genetic tools manipulating mycobiont has hindered studies on genetic mechanisms underpinning lichen biology. Thus, we established an *Agrobacterium tumefaciens*-mediated transformation (ATMT) system for genetic transformation of a mycobiont isolated from *Cladonia macilenta*. A set of combinations of ATMT conditions, such as input biomass of mycobiont, co-cultivation period with *Agrobacterium* cells, and incubation temperature, were tested to identify an optimized ATMT condition for the *C. macilenta* mycobiont. As a result, more than 10 days of co-cultivation period and at least 2 mg of input biomass of the mycobiont were recommended for an efficient ATMT, owing to extremely slow growth rate of mycobionts in general. Moreover, we examined T-DNA copy number variation in a total of 180 transformants and found that 88% of the transformants had a single copy T-DNA insertion. To identify precise T-DNA insertion sites that interrupt gene function in *C. macilenta*, we performed TAIL-PCR analyses for selected transformants. A hypothetical gene encoding ankyrin repeats at its C-terminus was interrupted by T-DNA insertion in a transformant producing dark-brown colored pigment. Although the identification of the pigment awaits further investigation, this proof-of-concept study demonstrated the feasibility of use of ATMT in construction of a random T-DNA insertion mutant library in mycobionts for studying genetic mechanisms behind the lichen symbiosis, stress tolerance, and secondary metabolite biosynthesis.

## 1. Introduction

Lichens can be defined as intimate symbioses between fungi and phototrophic partners, such as unicellular green algae and/or cyanobacteria, as the main symbionts. Lichens can thrive in harsh environments, often withstanding extreme heat, desiccation, or cold [1,2]. Moreover, some experiments demonstrated that lichens can survive in space, as well as in a simulation chamber that mimics an environmental condition of Mars [3,4]. About 20% of all known fungi are thought to have been lichenized and nearly 98% of them belong to the phylum Ascomycota [5,6,7]. As much as their species diversity, lichens produce a variety of secondary metabolites [8] that exhibit antitumor [9,10,11], antimicrobial [12,13,14,15,16,17], anti-inflammatory [18,19,20], and antioxidant activities [13,14,17,19], most of these bioactive compounds are considered to be originated from mycobionts. However, there have been a very few studies focusing on the ecological roles of these lichen secondary metabolites; some studies provide evidence that lichen secondary metabolites have contributed to adapting to harsher habitats [21] and deterring herbivores [22,23].

Mycobionts of many lichens have been isolated and grown separately in axenic culture. Despite the fascinating lichen biology, genetic studies on lichens has been limited, largely due to a paucity of genetic information and lack of molecular tools for manipulating mycobionts recalcitrant to genetic transformation. Polyethylene glycol-mediated introduction of DNA to protoplasts has been widely used in fungal transformation [24,25,26]. However, genetic transformation using protoplasts has not been successful, using mycobionts that generally exhibit extremely slow growth and form dense hyphal matrix resistant to cell wall degrading enzymes [27,28]. *Agrobacterium tumefaciens*-mediated transformation (ATMT) has been used to introduce and integrate T-DNA into random genomic loci in phylogenetically diverse fungi, such as *Saccharomyces cerevisiae* [29], several filamentous fungi [30,31,32,33] and a lichen-forming fungus of yeast form, *Umbilicaria muhlenbergii* [34]. The use of ATMT with different fungal tissues, such as spores and mycelia, can provide an alternative means to conduct genetic studies in mycobionts that do not readily form protoplasts [30,33,35].

**Figure 1:**
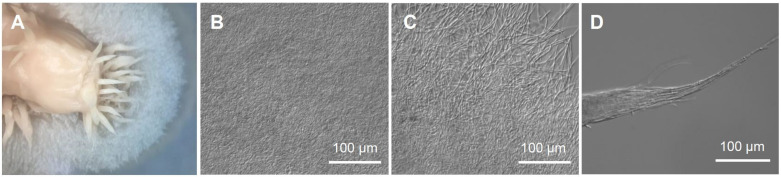
Colony morphology of a mycobiont isolated from *Cladonia macilenta*. (**A**) A front view of the colony of the *C. macilenta* mycobiont showing filamentous growth and biofilm formation on MEA media, (**B**) a central region of the colony comprised of dense hyphal matrix (i.e., biofilm), (**C**) a growing front of the colony, (**D**) a tip of protrusion from the colony center where the initial agar plug was placed for the culture.

The genus *Cladonia* with worldwide distribution belongs to the fungal class Lecanoromycetes that includes 70% of the known lichens [36]. The genus *Cladonia* has been a model for the laboratory reconstitution of mycobionts and phototrophic partners into developed lichen thalli [37,38,39,40]. We obtained a high-quality genome sequence of a mycobiont isolated from *Cladonia macilenta* Hoffm. to identify biosynthetic genes involved in production of biruloquinone, an acetylcholinesterase inhibitor [41], and well-known lichen substances found in nature, such as barbatic acid, squamatic acid, and didymic acid [42]. Moreover, *C. macilenta* can be a model mycobiont for studying in vitro reconstitution of lichen thalli, as the genome-sequenced mycobiont of *C. macilenta* exhibited a relatively fast growth rate (about 0.5 cm per month), compared to other mycobionts, and axenic cultures of the mycobiont on nutrient agar media formed biofilm with pin-like protrusion (see Figure 1), reminiscent of the lichen thalli of *C. macilenta* found in nature.

The objectives of this study were (i) to establish an ATMT method, testing different combinations of three factors in co-cultivation: input biomass of mycobiont, co-cultivation period with *Agrobacterium* cells, and temperature during incubation, (ii) to characterize resulting transformants, with respect to the number and insertion site of T-DNA in the genome of transformants, and (iii) to seek for the feasibility of use of the established ATMT for construction of a random T-DNA insertion mutant library for studying symbiosis, stress tolerance, and secondary metabolism in lichens.

## 2. Materials and Methods

### 2.1. Strains and Growth Conditions

A culture of the *C. macilenta* mycobiont (KoLRI021765) was obtained from the Korean Lichen and Allied Bioresource Center at Sunchon National University (Suncheon, Korea), which was stored in the form of agar plugs containing actively growing hyphae in 15% glycerol at –80 °C. The mycobiont was revitalized by shake culturing in 100 mL of malt extract broth (MEB, BD Biosciences, Baltimore, MD, USA) at 150 rpm and 15 °C in the dark. For transformation, 14-day-old culture was harvested by centrifugation at 5000 rpm for 5 min, and were washed three times with sterilized water. *Agrobacterium tumefaciens* AGL-1 strain that harbors the binary vector pSK1044 carrying a green fluorescent protein (GFP) [34] was used as a T-DNA donor for fungal transformation. *Agrobacterium tumefaciens* AGL-1 strain was cultured in Luria–Bertani media (LB, BD Biosciences, Baltimore, MD, USA) [43] at 28 °C.

### 2.2. Sensitivity of the C. macilenta Mycobiont to Hygromycin B

To evaluate sensitivity of the *C. macilenta* mycobiont against hygromycin B (Sigma-Aldrich, Munich, Germany), the mycobiont was grown on potato dextrose agar media (PDA, BD Biosciences Baltimore, MD, USA) supplemented with different concentrations of hygromycin (5, 10, and 15 µg/mL). Three independent cultures were incubated at 23 °C. The hyphal growth was observed every 3–5 days for 30 days until the mycobiont showed a significant growth.

### 2.3. Optimization of ATMT

To determine an optimal condition for ATMT, the *C. macilenta* mycobiont was transformed according to the established protocol [44] with minor modifications. The *Agrobaterium* strain AGL-1 carrying the binary vector pSK1044 was inoculated on LB media containing 50 µg/mL of kanamycin, at 28 °C, and was cultured overnight. Then, the culture was transferred to the induction media [29] containing 200 µM of acetosyringone (Sigma–Aldrich, Munich, Germany) and incubated for 6 h. The mycobiont was grown on malt extract agar (MEA, BD Biosciences Baltimore, MD, USA) for 30 days. To determine an optimum co-cultivation condition, examined were different input biomass of freshly harvested hyphae of the mycobiont (1, 2, and 5 mg), co-cultivation period of the mycobiont with the *Agrobaterium* strain AGL-1 (5, 10, and 15 days), and incubation temperature (22 °C, 25 °C, and 28 °C). This led to 27 different ATMT conditions, and each condition was tested with four replicate plates. One hundred microliters of *Agrobacterium* suspension (OD_600_ = 0.5) was mixed with 100 µL of ground mycobiont solution (10, 20, and 50 mg/mL) in sterilized water. The mixture was spread over Whatmann nitrocellulose filter paper (Roche Chemicals, Mannheim, Germany) covering the induction media containing 200 µM of acetosyringone in plates of 6 cm diameter. The plates were then subjected to different combinations of co-cultivation period and incubation temperature, prior to transferring the filter papers with the mycobiont and *Agrobaterium* cells to MEA selection media supplemented with 200 µg/mL of cefotaxime (Duchefa, Haarlem, The Netherlands) for killing bacterial cells, and 20 µg/mL hygromycin for selection of putative transformants of the mycobiont. Approximately one month later, mycobiont colonies in each plate were counted for statistical analysis.

### 2.4. Statistical Analysis

A three-way Analysis of Variance (ANOVA) was conducted to determine the effects of input biomass, co-cultivation period, and incubation temperature on transformation efficiency. Residual analyses were performed to test for the two primary assumptions for ANOVA test: normality and homogeneity of variances. Normality was assessed using Shapiro-Wilk’s normality test and homogeneity of variances was assessed by Levene’s test. Our original dataset violated the normality assumption (*p*-value = 0.002) and marginally satisfied the homogeneity of variances assumption (*p*-value = 0.052). We reasoned that this was due to extremely low transformation efficiency in the conditions using low input biomass of the mycobiont (1 mg). After excluding these conditions, the dataset satisfied both normality (*p*-values = 0.051) and homogeneity of variances (*p*-value = 0.683) assumption. Therefore, we performed ANOVA analysis only with two levels (2 and 5 mg) for the input biomass. Pairwise multiple comparisons were run for different co-cultivation periods, with a Bonferroni correction applied. All statistical analyses were performed using an R package *rstatix* (version 0.7.0).

### 2.5. Genomic DNA Extraction

Putative transformants and the wild-type strain of the *C. macilenta* mycobiont were grown in 30 mL of MEB medium at 15 °C in an orbital shaker (200 rpm) for 7 days for genomic DNA extraction. The mycobiont was harvested and ground into a fine powder under liquid nitrogen. DNA was extracted using DNAeasy mini kit, according to the manufacturer’s instruction (Qiagen, Valencia, CA, USA).

### 2.6. Real-Time PCR Conditions for T-DNA Copy Number Variation Analysis

Glyceraldehyde-3-phosphate dehydrogenase gene (*G3PD*) was used as a single-copy reference gene, and hygromycin phosphotransferase gene (*HPH*) was used to determine copy numbers of T-DNA integrated in the genome of transformants. Primers for *HPH*, and *G3PD* genes were designed to have similar melting temperatures of about 50–60 °C. The primer sequences were as follows: *G3PD*–fwd (GAAGGCCAAGGCTCACTTGAAG), *G3PD*–rev (GCAAGACAGTTCGTTGTGCATG), *HPH*–fwd (GGTGTCACGTTGCAAGACCTG), and *HPH*–rev (CGCCATGTAGTGTATTGACCG). Real-time PCR analysis was performed using the Bio-Rad CFX96 instrument (Bio-Rad Laboratories, Hercules, CA, USA). Real-time PCR mixtures were prepared as follows: 1.5 µL (10 pmol/µL) of each primer, 5 µL iQ^TM^ SYBR^®^ Green supermix (Bio-Rad Laboratories, Hercules, CA, USA), 2 µL of DNA (12.5 ng/µL) of transformant in a total reaction volume of 10 µL. The real-time PCR amplification was initiated with a 3 min step at 95 °C followed by 40 cycles of 10 s at 95 °C and 30 s at 55 °C. After the amplification, a melting curve analysis was performed with a temperature gradient of 0.5 °C from 65 °C to 95 °C for 5 s. Reactions were performed three times for establishing standard curves and copy number variation analysis. The averaged Ct values for replicates were used for the T-DNA copy number estimation.

### 2.7. Relative Quantification of T-DNA Copy Number

To determine copy number of T-DNA, the single-copy reference gene *G3PD* was quantified in parallel with *HPH* as a proxy for the T-DNA inserted in transformants. The relative quantification of the copy number was performed according to the 2^–ΔΔCt^ method [45], where:

ΔΔCt = ΔCt of transformants − ΔCt of a calibrator strain.

A strain (CmT-13) with one copy of *G3PD* and *HPH* was served as a calibrator strain. Since the measured fluorescence intensity is correlated with the numbers of integrated *HPH* in transformants, the copy number of *HPH* can then be calculated using, the equation below:

Copy number *_HPH_* = (1 + *E*)^−ΔΔCt^,

where, *E* is the PCR efficiency [46].

### 2.8. TAIL-PCR and Sequencing

Thermal asymmetry interlaced-PCR (TAIL-PCR) was used to identify the T-DNA insertion site in transformants, with minor modification [47]. T-DNA right border-specific primers (RB) and arbitrary degenerate (AD) primers were listed in Supplementary Table S1. The five AD primers (AD1, AD2, AD3, AD4 and AD6) were equally mixed and used in the first, second, and third round of TAIL-PCRs with RB1, RB2, and RB3 primers, respectively, at 2 mM in each reaction containing about 20 ng of gDNA of transformants. The resulting PCR products from the second and third round TAIL-PCRs were sent for sequencing at Macrogen Co. (Macrogen Co., Daejeon, Korea).

### 2.9. Microscopy

To confirm the expression of GFP in transformants, mycelia of randomly chosen transformants were mount on a slide glass and observed using a Zeiss Axio Imager A1 fluorescence microscope (Carl Zeiss, Oberkochen, Germany). Images were captured using differential interference contrast (DIC) and GFP filter sets.

### 2.10. Data Availability

The sequence of the four genes (Cma_08524, Cma_01716, Cma_02877, and Cma_09655) disrupted by T-DNA has been submitted to GenBank and may be found under accession number MW715785–MW715788. The genome assembly of the *C. macilenta* mycobiont was downloaded from the NCBI database (accession: GCA_000444155.1) and the whole genome annotation will be available from the corresponding authors upon request.

## 3. Results

### 3.1. The Mycobiont of C. macilienta

Lichen thalli of *C. macilenta* was collected from rotten bark at Mt. Cangshan (26°31′52.7″ N, 100°02′14.0″ E), Yunnan Province, China, in 2005. A voucher specimen (Institutional code: KoLRI003786) was deposited at the Korean Lichen Research Institute, Sunchon National University, South Korea. Mycobionts of *C. macilenta* were isolated by the ascospore discharge method [48]. The genome-sequenced mycobiont (KoLRI021765) [49] was grown on MEA for 4 months. The colonies of the mycobiont were formed (2–2.5 cm in diameter), and biofilm formation was observed in the central region of the colonies where initial agar plugs had been placed for culture (Figure 1). It is notable that protrusion of pin-like structures from the central biofilm was observed, somewhat resembling the natural thalli of *C. macilenta*, albeit these structures did not develop further (Figure 1A,D). Prior to transformation, the sensitivity of the *C. macilenta* mycobiont against hygromycin was evaluated to determine a minimal concentration of the antibiotics for the selection of transformants having T-DNA insertion. The growth of the *C. macilenta* mycobiont was significantly inhibited by 15 µg/mL hygromycin (Supplementary Figure S1). Therefore, we used 20 µg/mL of hygromycin for the selection of transformants.

### 3.2. Optimization of ATMT Method for C. macilienta

To determine an optimum condition for ATMT, we examined the effects of (i) input biomass of the mycobiont, (ii) co-cultivation period of the mycobiont with the *Agrobaterium* strain, and (iii) incubation temperature during co-cultivation. The *C. macilenta* mycobiont was transformed with *Agrobaterium* strain AGL-1 that harbors the pSK1044 plasmid carrying *GFP* and *HPH* as a selection marker. Putative transformants resistant to hygromycin were grown and counted approximately one month after the transfer to the selection media.

**Figure 2:**
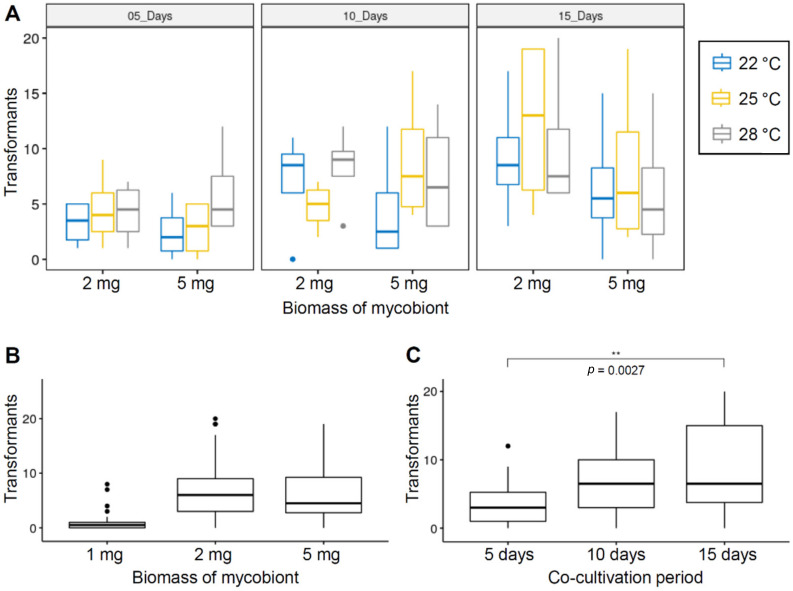
Optimization of ATMT of a lichen-forming fungus *C. macilenta*. (**A**) Box plots showing the number of transformants obtained, using different combinations of input biomass of the mycobiont, co-cultivation period and incubation temperature. Boxes indicate the median, interquartile range between the 25th and 75th percentiles, and whiskers indicate 1.5 interquartile range. Outliers are indicated by circles. (**B**) Effects of the input biomass of the mycobiont, (**C**) Effects of co-cultivation period in transformation efficiency. *n* = 36.

First, we investigated any possible interactions between the above-mentioned effects by a three-way ANOVA analysis. No interaction between the effects was found, and only co-cultivation period of the mycobiont with *Agrobaterium* have an influence on transformation efficiency (Table 1; Figure 2A). Since the original dataset including data for the low input biomass of the mycobiont (1 mg) violated the normality assumption for statistical analysis (see Materials and methods), we excluded this condition from the three-way ANOVA. Nevertheless, it is clear that transformation efficiency was significantly higher in the conditions using more biomass inputs (2 or 5 mg) than in the condition using the low input biomass (Figure 2B). There was a statistically significant effect of co-cultivation period, *F*(2, 54) = 5.522, *p*-value = 0.007 (Table 1). Pairwise comparisons between the co-cultivation period (5, 10, and 15 days) indicated a significant difference in transformation efficiency between 5 days and 15 days of co-cultivation period (Figure 2C).

### 3.3. T-DNA Copy Number Variation of Transformants

**Figure 3:**
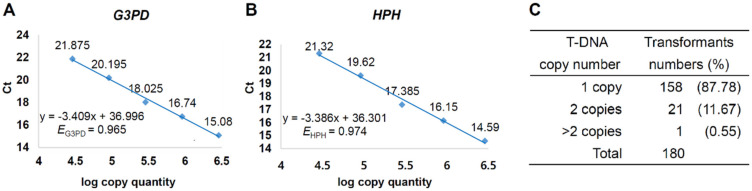
T-DNA copy number of *C. macilenta* transformants. Standard curves of (**A**) glyceraldehyde-3-phosphate dehydrogenase gene (*G3PD*) as a single-copy reference gene and (**B**) hygromycin phosphotransferase (*HPH*) as a proxy for T-DNA insertion event. (**C**) T-DNA copy number of *C. macilenta* transformants.

We selected 180 putative transformants resistant to hygromycin and determined their T-DNA copy number by real-time PCR analyses. Given the *Cladonia macilenta* genome size of 37.1 Mbp [49], 24,585 copies of the genome were estimated to be present in 1 ng of haploid *Cladonia macilenta* genomic DNA, according to the copy quantity equation [46]. The standard curves for a calibrator strain (CmT-13) were plotted by log-transformed copy quantity and Ct values of *G3PD* (reference gene) and *HPH* (target gene) in real-time PCR analyses, covering a copy quantity range from 2.9 × 10^4^ to 2.9 × 10^6^ copies (Figure 3A,B). The PCR efficiency was calculated from the slope of each standard curve following the equation *E* = 10^−1/slope^ − 1 [46], with the PCR efficiency of 0.965 and 0.974 for *G3PD* and *HPH*, respectively. These high PCR efficiency (>0.96) satisfied a prerequisite for calculations of gene copy number using the relative 2^−^^ΔΔCt^ method [50]. Based on the standard curves, T-DNA copy number of 180 transformants was determined; 88% of transformants were confirmed to have a single copy T-DNA, and 12% of transformants had two or more than two copies of T-DNAs inserted in the genomes (Figure 3C).

### 3.4. Identification of T-DNA Insertion Sites

**Figure 4:**
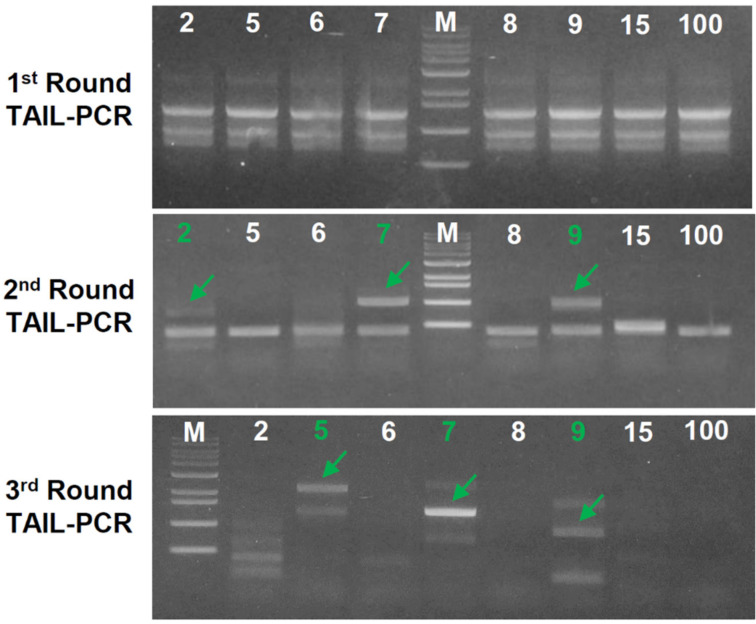
An example of TAIL-PCR analyses. Agarose gels showing PCR products from the first, second, and third rounds of TAIL-PCR analyses for eight selected transformants (numbers). Arrows indicate successful PCR amplification of a flanking region of T-DNA in the respective transformants (CmT-2, CmT-5, CmT-7 and CmT-9). M, DNA ladder.

To identify genomic loci where T-DNA was inserted in transformants, we performed TAIL-PCR analyses and subsequent Sanger sequencing of TAIL-PCR products. Among 158 transformants that have a single-copy T-DNA insertion, we selected 30 transformants for TAIL-PCR analyses. PCR products from the first round TAIL-PCRs displayed similar band patterns, whereas more specific bands appeared in the second round and third round TAIL-PCRs (Figure 4). Thus, we sequenced the PCR amplicons resulted from the second round and third round TAIL-PCRs, and obtained flanking sequences of the right border of the inserted T-DNA from 12 transformants (Table 2). To locate T-DNA insertion sites, flanking sequences of the T-DNA right border identified by TAIL-PCR analyses were mapped to the genome of *C. macilenta* by BLAST search. We found that T-DNAs in four transformants (CmT-2, CmT-81, CmT-82, and CmT-83) interrupted protein-coding genes, whiles T-DNAs were inserted in intergenic regions in the other eight transformants (Table 2).

### 3.5. Phenotyping of C. macilenta Transformants

**Figure 5:**
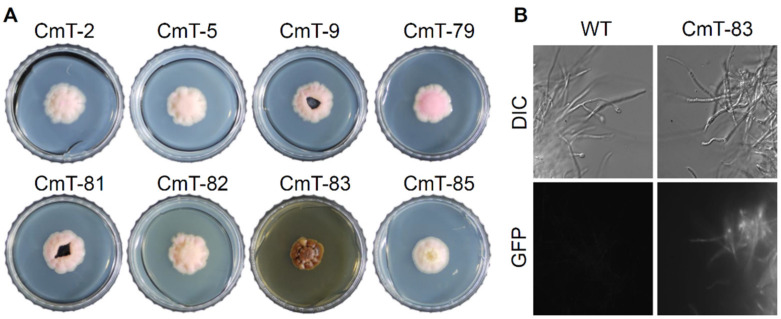
Phenotyping of *C. macilenta* transformants generated via ATMT. (**A**) Representative images of *C. macilenta* transformants growing on MEA. Note that dark brown pigment accumulated in the CmT-83 strain, (**B**) Microscopic images of *C. macilenta* wild-type (WT), and the CmT-83 strain expressing GFP. DIC, differential interference contrast image; GFP, fluorescence image.

Phenotype screening of transformants led to the identification of a putative mutant (CmT-83) differing from the wild-type in colony color and morphology on MEA media (Figure 5A). Compared to the pinkish (sometimes whitish) colony of other transformants exhibiting a similar colony morphology with the wild-type, the colony of the CmT-83 strain was heavily pigmented (Figure 5A). This dark-brown colored, water-soluble pigment accumulated in the media. We attempted to extract and identify this pigment, however the chemical profile of the culture extract of the CmT-83 strain showed no appreciable metabolite production in HPLC analysis (Supplementary Figure S2).

The expression of GFP in the colony of the CmT-83 strain further confirmed T-DNA insertion into the genome (Figure 5B). TAIL-PCR identified T-DNA in the CmT-83 was inserted 66 bp upstream of the start codon of the Cma_09655 gene encoding a hypothetical protein harboring 14 copies of ankyrin repeats at its C terminal region (Table 2). A BLAST search with the deduced 1620 amino acids sequence of the Cma_09655 gene showed only a few significant hits to genes in lichenized and non-lichenized fungi in the NCBI database (as of February 2021), showing 31–46% sequence identity and query coverage greater than 90%, suggesting divergent roles of these uncharacterized proteins in fungi.

## 4. Discussion

A paucity of genetic information and lack of molecular tools manipulating lichen-forming fungi recalcitrant to genetic transformation have hindered genetic studies on lichens. This study presents, for the first time, development and optimization of an ATMT system in lichen-forming fungi of filamentous growth form. This ATMT system will provide lichenologists with unique opportunities to study the genetic mechanisms behind lichen symbiosis, stress tolerance, and secondary metabolite biosynthesis.

Some studies showed that the efficiency of ATMT is affected by several factors in fungi, such as ratio of fungal biomass/*Agrobacterium* cells and growth conditions during co-cultivation [29,30,44,51,52]. For the optimization of ATMT conditions, a longer co-cultivation period (15 days) yielded significantly more transformants than a shorter co-cultivation period (five days). However, longer periods of co-cultivation may also cause multiple events of T-DNA insertion into the genomes of mycobionts, owing to prolonged contact with *Agrobacterium* cells. Insertion of T-DNA at different genomic loci would hamper identification of genes responsible for the observed phenotypic difference in transformants. Our data showed that transformation efficiency of the 10 days’ co-cultivation period was nearly identical to that of the 15 days’ co-cultivation period. Therefore, for mycobionts showing extremely slow growth rate, the 10 days’ co-cultivation period (or slightly longer period) may be recommended for an efficient ATMT, minimizing multiple T-DNA insertion events. Moreover, our data showed that the amount of input biomass had a great impact on transformation efficiency. We obtained only a very few transformants in the conditions using the low input biomass of the mycobiont (1 mg). The increase of input biomass from 1 mg to 2 mg resulted in a higher transformation efficiency. Therefore, we set the minimum amount of the mycobiont biomass to 2 mg for future experiments.

We analyzed T-DNA copy number variations in 180 transformants, using a real-time PCR analysis that was reliable and much faster than using standard southern blot analysis [50]. The house-keeping gene *G3PD* in *C. macilenta*, as a single-copy reference gene, was suitable for copy quantity estimation in a given genomic DNA. Moreover, the primers set we designed for *G3PD* and *HPH* amplification in real-time PCR analyses showed high PCR efficiency that is a prerequisite for a real-time PCR-based copy number calculation [45]. The frequency of transformants that contained a single copy T-DNA in *C. macilenta* was high (88%) and comparable to that observed in a lichenized fungus of yeast form [34] and a non-lichenized fungus, *Maganaporthe oryzae* [44].

Although the identification of dark brown pigment produced by the CmT-83 strain await further chemical investigation, the CmT-83 strain exhibited a dramatic change in its phenotype. The Cma_09655 gene interrupted by T-DNA in the CmT-83 strain encodes an ankyrin repeats-containing protein with unknown function. Ankyrin repeats are generally involved in protein–protein interactions and found in proteins of diverse function, such as transcription, cell cycle control, and signal transduction [53,54,55]. Therefore, it is conceivable that T-DNA insertion in the gene may have caused alteration in cellular signaling and expression of downstream genes involved in pigmentation, activating biosynthetic genes that would otherwise have been silenced.

In this study, we have developed an optimized ATMT system for mycobiont transformation. Thus far, this ATMT approach is the only available method for studying lichen-forming fungi, and may be applied to other mycobionts as well. Construction of a random T-DNA insertion library using the established ATMT method will enable us to conduct genetic screening and characterization of novel genes that are important for lichen symbiosis, stress tolerance, and secondary metabolite biosynthesis.

## Figures and Tables

**Table 1:** A three-way ANOVA on the number of transformants obtained in different combinations of (i) input biomass of the *C*. *macilenta* mycobiont, (ii) co-cultivation period with the *Agrobacterium* strain AGL-1, and (iii) incubation temperature. * *p*-value < 0.01.

Parameter	d.f.	*F* Statistics	*p*-Value	GES ^1^
Biomass input (i)	1	0.980	0.327	0.018
Co-cultivation period (ii)	2	5.522	0.007 *	0.170
Incubation temperature (iii)	2	0.671	0.515	0.024
(i):(ii)	2	1.069	0.351	0.038
(i):(iii)	2	0.131	0.878	0.005
(ii):(iii)	4	0.297	0.879	0.022
(i):(ii):(iii)	4	0.551	0.699	0.039

^1^ GES: generalized eta squared (effect size), measuring the proportion of the variability in the number of transformants generated.

**Table 2:** T-DNA insertion sites in transformants of the *Cladonia macilenta mycobiont*.

Strain	Flanking Sequence of T-DNA ^1^	Length ^2^ (Identity)	Insertion Site ^3^	Gene ID	Pfam Domain (*E*-Value)
CmT-2	ATGATCATAGAAAGGATGCC	134 (100)	17:318521	Cma_08524	no domain found
CmT-5	CAGGACGTCGATTGTAGCAC	149 (99)	5:324280	intergenic	-
CmT-7	CTGGAGGAGAATCAGGAGGT	237 (100)	2:2003077	intergenic	-
CmT-9	CGGCCGGGGAAAACCGTTCG	133 (100)	15:985193	intergenic	-
CmT-41	TCCGCTTTTTGGCAGGCTGC	142 (99)	21:390480	intergenic	-
CmT-45	AAGGAAACCTTACGCTAGTG	130 (100)	0:1782331	intergenic	-
CmT-79	TGGAGGGATGATCTCTATGG	105 (100)	16:437828	intergenic	-
CmT-81	ATTTTTTGTCCTGTCTGAGG	61 (100)	2:1031916	Cma_01716	PF00364 (7.9 × 10^−16^)
CmT-82	TAGGAAATCAATGGGTTAAG	527 (100)	4:843652	Cma_02877	PF01812 (1.5 × 10^−31^)
CmT-83	GTTCACCCCTCTTCGCAATA	642 (100)	20:650595	Cma_09655	PF12796 (3.8 × 10^−23^)
CmT-85	TAGCGGATGCTTTAGGCGAA	88 (100)	14:378660	intergenic	-
CmT-86	CCTAGAGTAAGGTAGGTATG	126 (100)	20:271306	intergenic	-

^1^ Only the first twenty nucleotides were shown. ^2^ Length of flanking region of the right border of T-DNA identified by TAIL-PCR analyses. ^3^ Numbers before and after colon refer to scaffolds and the position of nucleotide where T-DNA was inserted in scaffolds, respectively.

## Data Availability

Not applicable.

## Supplementary Materials

The following are available online at https://www.mdpi.com/article/10.3390/jof7040252/s1, Figure S1: Sensitivity of the *C*. *macilenta* mycobiont to hygromycin; Figure S2: HPLC profiles of culture extracts of (i) wild type (WT) *Cladonia macilenta* and (ii) the CmT-83 strain; Table S1: Primer used for TAIL-PCR analysis.
