# Supplementary material for: Establishment of Agrobacterium tumefaciens-Mediated Transformation of Cladonia macilenta, a Model Lichen-Forming Fungus
Source: J Fungi (Basel). 2021 Mar 26;7(4):252. doi: 10.3390/jof7040252 (PMC8065847; doi:10.3390/jof7040252)
Supplement: Supplementary file 1 [file jof-07-00252-s001.pdf]

Supplementary Information

Establishment of *Agrobacterium tumefaciens*-Mediated Transformation of *Cladonia macilenta*, a model lichen-forming fungus

by

Rundong Liu, Wonyong Kim, Jaycee Augusto Paguirigan, Min-Hye Jeong, and Jae-Seoun Hur

Korean Lichen Research Institute, Sunchon National University, Suncheon, 57922, South Korea

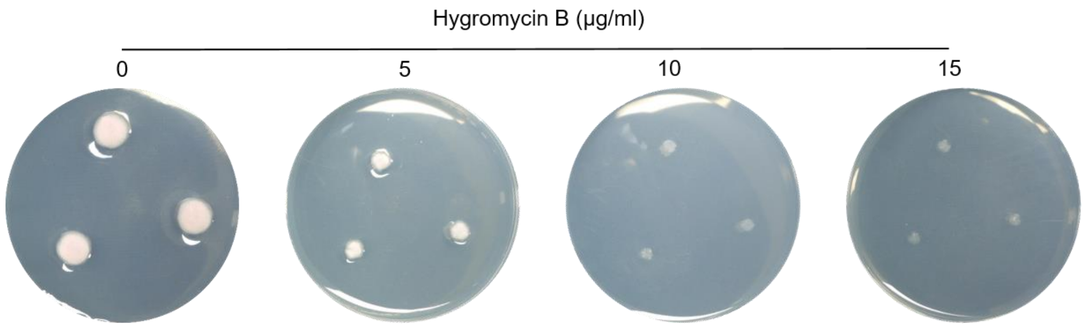

Supplementary Figure S1. Sensitivity of the *C. macilenta* mycobiont to hygromycin. Images of mycobionts growing on PDA containing different concentrations of hygromycin B were taken one month after culture.

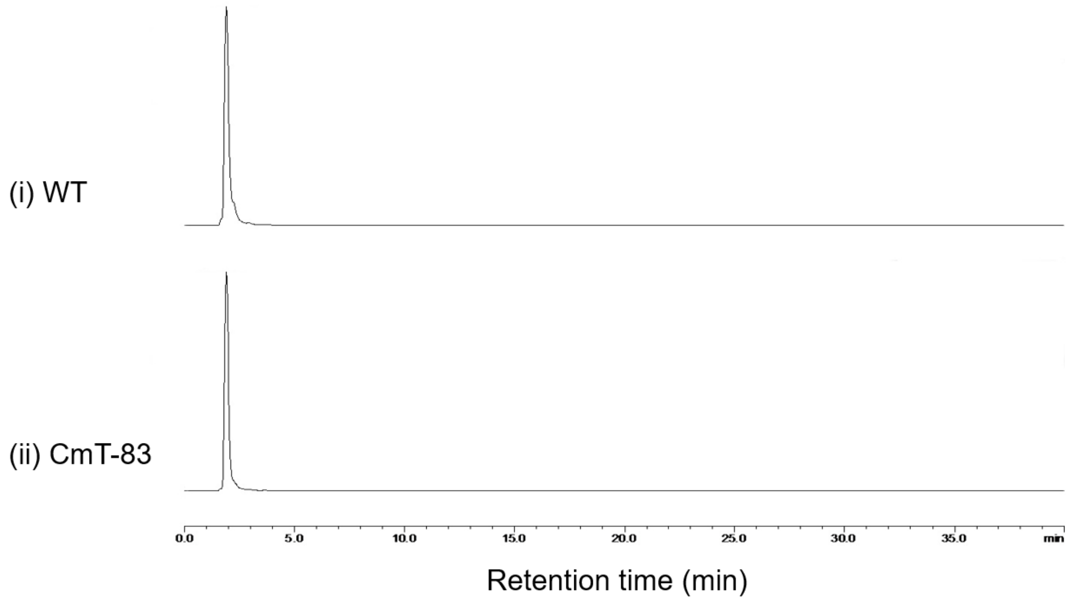

Supplementary Figure S2. HPLC profiles of culture extracts of (i) wild type (WT) *Cladonia macilenta* and (ii) the CmT-83 strain. The major peak appeared at 2 min is for acetone. The WT and CmT-83 strains were cultured in 500 ml flasks containing 100 ml MEB media for two and a half months. The cultures were harvested and extracted three times with 100 ml EtOAc. The EtOAc fractions were separated by funnels and subjected to dryness. The crude extracts were dissolved in 300 μl acetone transferred into a 2 ml microcentrifuge tube, and filtered through 0.4 mm syringe membrane before injection to HPLC. A high performance liquid chromatographic (HPLC, Prominence Modular HPLC LC-20A; Shimadzu, Kyoto, Japan) method was used for the detection of secondary metabolites. The culture extracts were analyzed on a YMC-Pack ODS-A column (length 150 × internal diameter 4.6 mm, particle size 5 um, pore diameter 12 nm, YMC Co., Ltd., Kyoto, Japan) at 40°C. Secondary metabolites were monitored by a diode array UV detector (SPD-M20A) at 254 nm. Solvent system uses pump B (MeOH: H_2O: Phosphoric acid = 80:20:1). Flow rate was 1.0 ml per min with a total time 40 min.

Supplementary Table S1. Primer used for TAIL-PCR analysis

Primers	Sequence (5'-3')	Description
RB1	GGCACTGGCCGTCGTTTTACAAC	T-DNA right border primer
RB2	CTGGCGTAATAGCGAAGAGG	T-DNA right border primer
RB3	CCCTTCCCAACAGTTGCGCA	T-DNA right border primer
RB4	GAATGGCGAATGCTAGAGCAG	T-DNA right border primer
AD1	AGWGNAGWANCAWAGG	Arbitrary degenerate primer
AD2	WAGTGNAGWANCANAGA	Arbitrary degenerate primer
AD3	WAGTGNAGWANCANGTT	Arbitrary degenerate primer
AD4	WAGTGNAGWANCANGAA	Arbitrary degenerate primer
AD6	WGTGNAGWANCANAGA	Arbitrary degenerate primer
